# Trolls Without Borders: A Cross-Cultural Examination of Victim Reactions to Verbal and Silent Aggression Online

**DOI:** 10.3389/fpsyg.2021.549955

**Published:** 2021-06-01

**Authors:** Christine Linda Cook, Juliette Schaafsma, Marjolijn L. Antheunis, Suleman Shahid, Jih-Hsuan Tammy Lin, Hanne W. Nijtmans

**Affiliations:** ^1^Department of Information Sciences, New Jersey Institute of Technology, Newark, NJ, United States; ^2^Department of Communication and Cognition, Tilburg University, Tilburg, Netherlands; ^3^Syed Babar Ali School of Science and Engineering, Lahore University of Management Sciences, Lahore, Pakistan; ^4^Department of Advertising, National Chengchi University, Taiwan Institute for Governance and Communication Research, Taipei, Taiwan; ^5^Department of American Studies, University of Groningen, Groningen, Netherlands

**Keywords:** trolling interactions, flaming, ostracism, experiment, Cyberball, cross-cultural comparisons, honor, face

## Abstract

Trolling—the online exploitation of website, chat, or game mechanics at another user's expense—can and does take place all over cyberspace. It can take myriad forms, as well—some verbal, like trash-talking an opponent in a game, and some silent, like refusing to include a new player in a team effort during an in-game quest. However, despite this variety, there are few to no studies comparing the effects of these differing trolling types on victims. In addition, no study has yet taken into account users' offline cultural context and norms into the trolling victim experience. To fill this gap in the literature, the present study put participants from three culturally-distinct countries—Pakistan, Taiwan, and the Netherlands—in a simulated trolling interaction using the Cyberball game. Participants were either flamed (read: harshly insulted) or ostracized by a member of their own cultural group (ingroup) or a minority member (outgroup), and the participants' emotional responses, behavioral intentions toward the other players, and messages sent during the game were taken as indicators of their response to the trolling. Results showed that our Taiwanese sample used the most reactive aggression when trolled and our Dutch sample was the most passive. In addition, ostracism generally produced the desire to repair relationships, irrespective of cultural context, and perpetrator culture (ingroup or outgroup) only produced an effect in the behavioral intentions of our Pakistani sample. Overall, it would appear that online and offline culture interact to produce the variety of responses to trolling seen in extant literature. Additional implications for future research into computer-mediated communication and online aggression are also discussed.

## Introduction

Around the world, anyone who uses a social network, comments on YouTube, or plays an online game even casually is at risk of experiencing online hostility, referred to as trolling (e.g., Buckels et al., 2014). In the online gaming world, experiencing trolling is a kind of rite of passage (Cook et al., 2018), and trolling behavior can take a myriad of forms. For example, “trash talking” is a commonly used technique whereby a player insults or abuses another player, often with the intent to annoy him or her or to derail the game (e.g., Cook et al., 2018). In other instances, the trolling behavior is much more subtle, such as a player ignoring a teammate's cries for assistance, or purposely lengthening the game by refusing to take their turn, known as “bad manner” in gaming communities (Arjoranta and Siitonen, 2018). In such cases, the intent of the aggressor is often either hidden, or at least more ambiguous.

How do people react to these different forms of online aggression? At first glance, it may appear that especially overt forms of aggression, such as insults—called flaming in the online context (O'Sullivan and Flanagin, 2003)—should be particularly aversive, as they form a direct threat to people's self-esteem and reputation, to which they typically respond with embarrassment or anger (e.g., Liu et al., 2018). One could also make the case, however, that more covert, non-verbal forms of aggression, such as ostracism should be equally or perhaps even more aversive, as they threaten people's fundamental needs, such as their sense of belonging and their self-esteem, but also their sense of existence and recognition (e.g., Williams, 2009). Whereas, people do get some attention and recognition, albeit negative, when they are being insulted, ostracism sends the message that they are unworthy of attention at all (see James, 1950; Filipkowski and Smyth, 2012; Hartgerink et al., 2015). When people experience this, they have been shown to respond in a variety of ways. Although there is some evidence that they may, under certain circumstances, try to seek re-inclusion (e.g., Ouwerkerk et al., 2005), various studies also show that they can respond with anger and aggression (e.g., Hales and Williams, 2018) or even seek solitude (Leitner et al., 2014).

To our knowledge, however, there has been no or little research comparing people's reactions to verbal and non-verbal forms of online aggression and so, at present, it is not clear whether they result in similar or different responses. The main goal of the present study is to address this issue, by examining how people from different cultural contexts respond to being flamed or being ostracized by in-group or out-group members. We use a cross-cultural angle and rely on samples from differing contexts because cultures have different norms when it comes to responding to threats to the (social) self, and also have different norms about the need to maintain harmony and fit into the group, which could impact how people react to insults and ostracism (e.g., Bond et al., 1985). For instance, according to much of Cohen's and Nisbett's work (e.g., Cohen and Nisbett, 1997), in cultures where honor is more salient and where maintaining respect is a central virtue, people should respond quickly and even aggressively when their reputation is threatened with witnesses present, particularly when the perpetrator is not a member of their social group (Allpress et al., 2014; Anjum et al., 2019; Giner-Sorolla, 2019). Yet, in cultural settings where maintaining face or avoiding face loss and ingroup harmony are more important, people may be more likely to feel embarrassed by a flame and prefer to avoid confrontation, especially if the aggressor is an ingroup member (e.g., Lee et al., 2014).

To examine how people across different cultural contexts respond when flamed or ostracized, we conducted an experiment in Pakistan—which is generally considered an honor culture (see Anjum et al., 2019)—and Taiwan, which has been described as a face culture (see Ting-Toomey et al., 1991). As an additional comparison group, we also included participants from the Netherlands, where concerns about face and honor are likely to be less salient or prevalent, and where people theoretically develop a sense of self that is relatively insensitive to the influence of others (see Markus and Kitayama, 1991; Leung and Cohen, 2011). We were principally interested in how angry and how embarrassed or humiliated participants across these different settings would feel following insults or ostracism by ingroup or outgroup members, and whether they would be motivated to retaliate or would prefer to withdraw or to restore relationships instead.

## Theoretical Background

### Honor Concerns and Reactions to Verbal and Silent Aggression

As mentioned in the introduction, the present literature on how people respond to threats to the self would suggest that people from a culture in which honor is salient should be particularly sensitive to overt, verbal forms or aggression, such as flaming. Of particular importance in this regard is Leung and Cohen (2011) theory regarding how different cultures conceive of reputation as a concept. Although there are three conceptions according to this theory—dignity, honor, and face—honor is arguably the most researched in terms of its connection to aggression (see Nisbett and Cohen, 1996). Leung and Cohen (2011) describe honor as being a combination of how people see themselves and how society sees them: “honor must be claimed, and honor must be paid by others” (Leung and Cohen, 2011, p. 509). In other words, it is up to each person to both develop their own reputation (honor), and also to treat other people with the respect their honor deserves. When someone does not pay a person respect according to their honor, the victim loses their honor, and has to fight or punish the offender to regain it. This tendency to employ “reactive aggression” (Ang et al., 2014) following threats to one's honor has been shown with relative consistency in empirical work among cultures that conceive of reputation in this way. Cohen and Nisbett (1994), for example, found that men in the southern United States endorse violence when they are trying to defend their honor or the honor of their family, while Uskul and Cross (2018) found repeatedly that their Turkish participants were particularly likely to retaliate aggressively when they perceived a loss of honor via insult or accusation. These findings provide support for the idea that in a setting where honor is valued, the cultural norms are more likely to dictate that retaliation to regain honor is justified (e.g., Glick et al., 2016).

Yet, while members of honor-valuing cultures may react with reciprocated aggression to a flame due to the obvious insult to their honor, there is also reason to believe that they may be less likely to defend their honor when ostracized. Although neither aggression option in the present study is pleasant to experience as a victim, there is a sharp distinction between flaming and ostracism when it comes to the idea of insult. Flaming is far more direct, as it consists of verbal insults and hostility directed at the victim, often peppered with profanity and expressed with a liberal use of the caps lock button (O'Sullivan and Flanagin, 2003). When a person is being ostracized, however, the reason behind the ostracism is not expressed, and so the victims are left to their own devices when it comes to interpreting the hostility as insulting or otherwise (Williams, 2009). In short, ostracism is a form of aggression that can and often does hurt (see Williams et al., 2000; Williams, 2009), but it is not necessarily *insulting*. Empirical work suggests that in such situations when a direct insult is not perceived, people from honor-valuing cultures may actually prefer peaceful solutions to their conflicts (Harinck et al., 2013; Pfundmair et al., 2015). In Harinck et al. (2013) study, for instance, honor-valuing participants who were told to imagine being in a conflict, but not insulted, still agreed to work with the other party in the conflict to solve the issue together, contrary to the participants who were insulted. In Pfundmair et al. (2015) study, they also found that members of more collectivistic cultures—which honor-valuing cultures generally are (see Anjum et al., 2019)—do not share the aggressive intentions of dignity-valuing cultures when faced with ostracism. They do, however, experience ostracism more intensely than their more individualistic counterparts (Smith and Williams, 2004; Kimel et al., 2017), as they are more socially interdependent (Uskul and Over, 2017). This can, of course, vary based on individual differences like attachment style (Yaakobi and Williams, 2016a) and religious beliefs (Yaakobi, 2021)—but on the whole, extant literature would suggest that ostracism is an intensely negative experience for people from collectivist cultural contexts.

This social interdependence, alongside other theory and empirical work, would both suggest that people from honor-valuing cultures respond to ingroup and outgroup members differently (e.g., Cross et al., 2013). More specifically, there is reason to believe that in honor-valuing cultures, verbal aggression by outgroup members—operationalized in the present study as flaming—should result in anger and a stronger desire to retaliate than if the flaming was performed by an ingroup member. This difference is rooted in the beliefs in honor-valuing cultures that honor needs to be defended when threatened, and that honor is shared amongst ingroup members (Leung and Cohen, 2011). When a person retaliates against an aggressor, particularly when the aggressor is employing such an overt tactic as flaming (see Cook et al., 2018), they are fundamentally risking their relationship with that person. When the perpetrator is an outgroup member, there is no existing relationship to threaten, and so the maxim of defending one's reputation is free to be pursued by the honor-valuing victim (Leung and Cohen, 2011; Severance et al., 2013). However, when the perpetrator is an ingroup member, there is a critical pre-existing relationship that could be threatened by retaliating (Severance et al., 2013; Uskul and Over, 2014). When people aggress a close ingroup member in this kind of cultural context, they are risking their own social standing (Leung and Cohen, 2011; Severance et al., 2013). Thus, the risks associated with retaliating against an ingroup offender are likely to be judged too high, and so reactive aggression should theoretically be reserved for aggression originating from an outgroup perpetrator.

Silent forms of aggression, such as ostracism, however, should produce more embarrassment than anger at having caused an unknown offense leading to being ostracized, as well as a stronger tendency to engage in repairing the relationship when the perpetrator is an ingroup member, as opposed to an outgroup member. Part of this expectation comes from the fact that embarrassment is a negative, self-conscious emotion that is produced when a person's identity is being threatened in some way (Chen et al., 2020; Dasborough et al., 2020). Ostracism is a potential threat to a person's social identity, particularly when coming from an in-group member (e.g., Severance et al., 2013). This is likely to be amplified by honor-valuing cultures' emphasis on the ingroup (typically the family) and interconnectedness (Markus and Kitayama, 1991; Leung and Cohen, 2011; Severance et al., 2013). The ingroup is generally the person's source of reputation, and often of basic necessities like food and shelter (Severance et al., 2013). If a person is being flamed by an ingroup member, the connection to the ingroup is only being risked if they choose to retaliate. However, if people are being ostracized by their ingroup, then they are potentially losing not only their social standing and sense of belonging, but also their livelihood. Ostracism is also often used as a form of punishment in certain communities or populations (see Freedman et al., 2016; Hales et al., 2016; Poon and Chen, 2016), so they may be embarrassed at having done something to deserve this punishment. In such a situation, it is more commendable to ignore the offense to preserve honor and relationships (see Cross et al., 2013 for examples), but repairing the relationship would be even more desirable, as it could lead to the victim's reconnection to their source of security (see Severance et al., 2013). This hope for reconciliation within the in-group has been demonstrated repeatedly when it comes to negative self-conscious emotions (shame, guilt, and embarrassment), particularly when it comes to ingroup members witnessing other ingroup members transgress (Allpress et al., 2014; Giner-Sorolla, 2019). We can see also this in action in Uskul and Over (2014) study of farmers and herders, in which farmers—who are more dependent on the family unit for their sustenance—responded less negatively to being ostracized by strangers than herders, who typically depend on the patronage of strangers to survive. In essence, when an honor-valuing culture member's connection to the ingroup is being threatened by ostracism, they will theoretically try to repair that connection; when no such connection exists, as with the outgroup, they have no need to engage in said reparative actions.

However, it should be noted that most studies on honor-valuing cultures and aggression were conducted in face-to-face settings if it is a physical experiment or observational study (e.g., Cohen and Nisbett, 1997), or an imagined face-to-face setting if it is a survey or vignette study (e.g., van Osch et al., 2013). Although we recognize this difference between the present study and extant literature, we continue to base our expectations on what we do know, as very few studies have examined honor-valuing cultures in the online context. For instance, we expect that honor-valuing people will react with more anger to outgroup members than ingroup members that flame them (H1a). The two studies that do focus on honor concerns online—Günsoy et al. (2015), as well as Pearce and Vitak (2016)—results confirm that honor-valuing people are just as concerned about protecting both their own honor and the honor of their families on- and offline. Thus, even though we have far fewer bystanders in the present study than on social media, the idea of protecting one's honor should not be less salient than if we conducted the study offline. Much of extant literature also presents a different social distance between the ingroup members than the present study. While these studies, along with many offline studies (e.g., Severance et al., 2013), focus on close ingroup members like parents, the present study aims to simulate an average online trolling experience, which typically involves strangers (see Synnott et al., 2017; Cook et al., 2018). Although we do make a distinction between ingroup and outgroup, our ingroup—fellow students of the same University and nationality—is unlikely to be as important to our participants as their family members. However, again, there are surprisingly few studies that deal with the intersection of cultural values and tie strength, and those that do tend to focus on entrepreneurship and business (e.g., Ma et al., 2011; Johnson et al., 2013), or how social groups harness social media to build new ties and strengthen existing ones (e.g., Gonzales, 2017; Kwak and Kim, 2017; Verdery et al., 2018). Thus, although the literature is admittedly scant in an online context, what exists appears to support our hypotheses.

### Face Concerns and Reactions to Verbal and Silent Aggression

Just as existing literature predicts that members of honor cultures will respond differently to flaming and ostracism, it also predicts that members of face-valuing cultures will react to ostracism in much the same way as members of honor-valuing cultures, but will not retaliate when flamed. Unlike the construct of honor, face is exclusively an external evaluation of a person's worth, and thus cannot be gained, but can be easily lost (Leung and Cohen, 2011; Hashimoto and Yamagishi, 2013). This also means that in social interactions, it must be carefully preserved, and unlike honor, it cannot be regained via defense. To prevent the loss of face, researchers have posited that in cultures where face is valued as people's primary form of reputation, they will avoid conflict when possible, ignoring perceived slights and hostility in order to preserve the face of everyone involved (Hashimoto and Yamagishi, 2013). Empirically speaking, this is most evident in cyberbullying research, where students from countries in East Asia—often presented in cross-cultural studies as “face cultures”—will avoid confronting aggressors directly for fear of losing face. Instead, these students try to seek support from others after the fact, or report the aggressor to an authority figure privately (Li, 2008; Ma and Bellmore, 2016).

The tendency to avoid conflict to preserve face in any aversive situation—flaming or ostracism—has been empirically demonstrated in both positive and negative situations (e.g., Bresnahan et al., 2002; Peng and Tjosvold, 2011), meaning that it is considered equally reprehensible to seek and accept praise without demonstrating humility and to retaliate against an aggressor (Kim and Cohen, 2010; Lee et al., 2014). In essence, the literature suggests that members of face-valuing cultures are driven by a desire to avoid individual attention, positive or negative, in all social situations. This is contrary to honor-valuing cultures, where people would theoretically be more likely to defend their reputation when it is being directly threatened via insult. Whether a person is being praised or insulted in a face-valuing cultural context, they are unlikely to give a strong reaction, instead choosing to withdraw, as being too proud or being too aggressive would result in an irredeemable loss of face (Leung and Cohen, 2011).

It is important to note, however, that most face-valuing cultures share the ingroup-centric values common to most honor-valuing cultures (Severance et al., 2013; Anjum et al., 2019). As such, we expect the experience of being flamed by an ingroup member to be a more intensely negative experience for people from face-valuing cultures than the experience of being flamed by an outgroup member. Nevertheless, when people from a face-valuing culture believe that an ingroup member is flaming them—much like the case with honor-valuing culture members—retaliation may not be an option, as this would risk damaging the relationship with the ingroup (see Leung and Cohen, 2011; Severance et al., 2013). This may be compounded by the avoidance tendency found in many empirical studies on how people from face-valuing cultures respond to threats and aggression in general (see Hashimoto and Yamagishi, 2013, 2016). Theoretically, therefore, an ingroup perpetrator would put people from face-valuing cultures into a particularly uncomfortable position when being insulted, as their primary goal is to preserve face and fit in with the ingroup (see Severance et al., 2013). This would, according to Kitayama et al. (2006), lead to what they call negative engaging emotions, such as embarrassment or shame, as well as a desire to withdraw. Sadness has also been recorded as a possible response to ostracism, in particular (Kimel et al., 2017). Although they may also want withdraw from conflict in the face of an outgroup member due to their conflict avoidance (see Hashimoto and Yamagishi, 2013, 2016), the same risk of being disconnected from the ingroup—their source of reputation and sometimes basic necessities (Severance et al., 2013)—does not exist to the same degree in this circumstance.

In the case of ostracism, we expect to see the same pattern with people from face-valuing cultures as we expect with honor-valuing cultures: an increase in attempts to repair the relationship when faced with an ingroup perpetrator when compared to an outgroup perpetrator. As is the theoretical case with flaming, ostracism inherently violates the collectivistic, ingroup-centric values of face-valuing culture members: preserving social harmony through fitting in and avoiding conflict (Peng and Tjosvold, 2011; Severance et al., 2013; Pfundmair et al., 2015). Just as is the theoretical case for people from honor-valuing cultures, being ostracized by an ingroup member adds an extra dimension of rejection for members of face-valuing cultures, as this means that they are being actively separated from their support network and source of reputation (see Severance et al., 2013). As in the case of honor-valuing people, this may not work in exactly the same way online with strangers as it does offline with family members. However, participants are aware that they are being recorded in-game, though personal anonymity is guaranteed; this should still elicit at least some face concerns, even if they are not as strong as if it was an in-person family-based situation.

Due to their inherent motivation to fit in and preserve harmony, although their preference would be to withdraw and avoid conflict, face-valuing culture members should be more likely to try and repair the relationship when ostracized by an ingroup member, restoring the harmony they allegedly prize (Hashimoto and Yamagishi, 2016). Again, this is the case with most cultures when it comes to ingroup transgressions (e.g., Allpress et al., 2014; Giner-Sorolla, 2019) and self-conscious emotions (Chen et al., 2020; Dasborough et al., 2020); this effect is simply theoretically amplified by the rejection-avoidance inherent to the cultural logic of face (Leung and Cohen, 2011; Hashimoto and Yamagishi, 2013, 2016). When an outgroup member is the perpetrator, like the situation with honor-valuing culture members, there is no pre-existing relationship to repair, and so they are not losing the vital connection to the ingroup (Severance et al., 2013). As such, there is no need to engage in relationship reparation with outgroup members and ignoring the offense and withdrawing should be enough to preserve the face of all parties involved (Markus and Kitayama, 1991; Leung and Cohen, 2011; Severance et al., 2013).

## The Present Study

As was mentioned in the introduction, the main goal of this study is to explore how people from different cultures respond to overt online aggression (flaming) and more covert forms of aggression (ostracism) by ingroup and outgroup members. To do this, we conducted a study across three different countries: Taiwan (representing face-valuing cultures), Pakistan (representing honor-valuing cultures), and the Netherlands to serve as a comparison country. These countries were selected because each had been previously used in extant literature as a representation of one of Leung and Cohen (2011) three cultural logics: face (Chien et al., 2018), honor (Anjum et al., 2019), and dignity (Ijzerman and Cohen, 2011). Because of this, we implicitly expected these countries to differ in terms of their people's self-construal (Pakistan and Taiwan having more interdependent people, and the Netherlands having more independent people; see Lee et al., 2014) and their concern for reputation [Pakistan having the most concern, followed by Taiwan, and finally the Netherlands; see Anjum et al. (2019) for a full discussion of how these cultural logics differ in these ways].

In our study, we examined three different types of responses to being either ostracized or flamed in the Cyberball game described in Williams et al. (2000) study: emotional responses, behavioral intentions, and actual behavioral responses. To our knowledge, this is the first study that examines the results of different types of online aggression across all three of our indicators, allowing us to capture nuances in the victim experience that were previously invisible. We were particularly interested in the differences between flaming and ostracism—overt and covert online aggression—in regards to how participants from the three cultural contexts felt (e.g., anger vs. embarrassment), as well as their behavioral intentions; which types of aggression elicit the desire to retaliate in which contexts, for example? We also recorded and coded every message sent by each participant as a record of their behavioral responses to either flaming or ostracism, depending on their assigned condition. In this way, we were able to see not only the practical results of our manipulation, but also how intention differs from action in trolling interactions.

Thus, we anticipate the following to occur in the present study:

**H1a**. When flamed, participants from cultures that value honor will report more anger and be more aggressive than when ostracized (in terms of their intentions and their behaviors), particularly when the perpetrator is an outgroup member as opposed to an ingroup member.

**H1b**. When ostracized, participants from cultures that value honor will be more likely to feel embarrassed (emotions) than when flamed, and will want to try to repair the relationship (intentions and behavior), particularly when the perpetrator is an ingroup member as opposed to an outgroup member.

**H2a**. When flamed, participants from cultures that value face will tend to feel embarrassed and to withdraw compared to when ostracized (in terms of their behavioral intentions and behaviors), particularly when faced with ingroup perpetrators as opposed to outgroup perpetrators.

**H2b**. When ostracized, participants from cultures primarily valuing face are also likely to feel embarrassed, particularly when ostracized by ingroup members, but they will also be more motivated to try to repair the relationship with them than when they are flamed.

## Method

### Participants and Design

We conducted the experiment among a sample of 451 participants across the three countries: Taiwan, Pakistan, and the Netherlands. Of these 451 original participants, there were errors saving the Cyberball data of 21, leaving us with 430 participants with completed data. Then, upon further inspection, we noticed that seven additional participants were below the age of 18, meaning they also had to be removed, leaving us with our final total of 423 participants. The Taiwanese sample consisted of 139 participants (108 women, 31 men) between the ages of 18 and 30 (*M* = 21.56, *SD* = 2.36), the majority of whom were highly educated (90), and the rest having obtained a medium level of education (49) according to UNESCO (2012). The Pakistani sample consisted of 149 participants (46 women, 103 men) between the ages of 18 and 41 (*M* = 22.73, *SD* = 2.95), their education levels evenly split between high (74) and medium (75). The Dutch sample consisted of 135 participants (94 women, 41 men) between the ages of 18 and 27 (*M* = 21.19, *SD* = 2.29), with the majority of these having obtained a low (77) or medium (49) level of education, and only a few (9) having obtained a high level of education [see UNESCO (2012) for full descriptions of the education levels here].

The study itself took a 3 (nationality: Taiwan, Pakistan, or Dutch) × 3 (types of trolling: flaming, ostracism, or control) × 2 (perpetrator group membership: ingroup or outgroup) experimental design. Participants were randomly assigned to the trolling and perpetrator group membership conditions using Qualtrics' built-in random participant assignment function. In order to be certain that expected country-level patterns did differ in our samples in the ways extant literature described (see Smith et al., 2017), all participants were assessed for individual self-construal and concern for reputation. University-aged students were selected because this is an age group that is likely to be exposed to trolling regularly (Cook et al., 2018). For our ingroup and outgroup manipulation, we chose to use minority groups within each country as our outgroup (Afghani in Pakistan, Filipino in Taiwan, and Moroccan in the Netherlands). Partially, this was because we wanted to be sure to have an effect, and using minority groups when assessing behavioral intentions and aggression had been successful in existing literature (e.g., Schaafsma and Williams, 2012). The study was approved by two separate institutional review boards: one at a mid-size University in Tilburg (whose assessment was also accepted for the Taiwanese portion of the study), and one at a large University in Pakistan.

### Procedure

In Taiwan (Taipei) and Pakistan (Lahore), participants were recruited via online advertisements in University fora and University-specific Facebook groups, as well as via snowball sampling. In the Netherlands (Tilburg), the majority of participants were recruited via a subject pool. Only when the subject pool was depleted were participants recruited using on-campus advertising. Except for the Dutch students who participated for course credit, participants were compensated with a small monetary token appropriate to each country.

Upon arrival in the lab, participants were told that the purpose of the study was to examine mental visualization across cultures (see Williams et al., 2000), and that the full session would consist of a pre-experiment questionnaire, a simple online game, and a post-experiment questionnaire. They were informed that elsewhere in the University, the same procedure was happening with two other participants with whom they would play an online game during the experiment. After giving their consent to participate, participants were asked to complete a questionnaire prior to starting the game with the other two participants. At this point, the research assistant left, and waited outside the room for the participant's knock, signaling that they were ready to begin the game. When the participant knocked, the researcher would re-enter the room, faking having received a text confirming that the other two participants (who are actually pre-programmed computer players) were ready to enter the game. They would then briefly review the mechanics of the game (how to type messages to other players and how to toss the ball) and leave the room again before the game began.

The game itself—Cyberball, a virtual ball toss game—was embedded into Qualtrics. This is a simulation of a game of catch (see for a more elaborate description, Williams et al., 2000). Participants each have a simple avatar who take turns tossing a ball back and forth between three or more players, at least some of whom are pre-programmed to behave in a certain way. In the present study, the three-player version was used, and the participant was the only human player. Each game consisted of 30 throws, and took ~3 min to play. Upon entering the game, participants received the same instructions (in their local language—Mandarin for Taiwan, English for Pakistan, and Dutch for the Netherlands, see Appendix B in Supplementary Material) to imagine that they were playing a real game of catch in a real park, engaging their senses as much as possible to create a detailed mental picture. They were asked to introduce themselves to the other players, and were informed that they could chat with the other players in game and were explained how to do so.

Upon entering the game, the two computer players would introduce themselves, giving their nationality (decrying their ingroup or outgroup status, depending on whether or not they shared the nationality of the participant) and a fake interest, either music or football. In Pakistan, for example, an ingroup perpetrator would introduce themselves as follows: “Hi! My name is Ahmed. I grew up here in Lahore. I'm a big fan of football!” Across all conditions, this would be the format, with the following names and outgroups substituted per country in the outgroup conditions: an Afghani named GulShar in Pakistan, a Pilipino named Danilo in Taiwan, and a Moroccan named Mohammed in the Netherlands. After this, the game would proceed depending on the participant's assigned condition. In the control conditions, there was no further chat from the computer players, and these were programmed to pass the ball randomly between each other and the human participant. In the flaming conditions, participants would be repeatedly insulted by Player 1, who would also periodically insult the other computer player. These insults, focusing on the player's childishness (e.g., “You play like a child”) or ineptitude, were pre-tested in each of the participating countries to ensure that they were insulting without being ethically dangerous, and equally offensive in each of the countries (see Appendix B in Supplementary Material). The ball was passed using the same randomized pattern as in the control conditions. In the ostracism conditions, there were no further messages sent by the computer players, but the ball never left the computer players' avatars; the human participant never had the opportunity to receive or pass the ball, although they were still able to use the chat function.

After the game, participants were redirected to a final questionnaire, which included the main dependent variables and manipulation checks. Once they had completed the questionnaire, the participant knocked on the door a final time and the research assistant re-entered the room. At this point, the assistant would perform a suspicion check by asking the participant what they thought the study was about and would then debrief and give the participant their participation fee (either a course credit or a monetary token, depending on the country of participation). This debrief consisted of the research assistant explaining the purpose and design of the study, including details regarding how the “other participants” were in fact pre-programmed computerized confederates. They were also careful to explain the random assignment procedure to ensure that no students felt that they were particularly selected to be either ostracized or flamed. After confirming that participants were unharmed and in an acceptable emotional condition, research assistants offered participants a pamphlet explaining trolling and cyberbullying, as well as providing local mental health resources available.

### Materials

All materials were administered in a local language: Mandarin for Taiwanese participants, English for Pakistani participants (the University's formal language was English) and Dutch for Dutch participants. In Pakistan, there was no need for translation, as the original language of all measures and scripts was English. For the Dutch and Mandarin editions, all materials were initially submitted for professional translation. After receiving these translations, teams of two to three bilinguals (English-Dutch or English-Mandarin) went over each item and made any adjustments to the language to make sure it corresponded to the original English. Finally, these versions were back-translated by other bilinguals, and final adjustments were made by the same team of bilinguals that performed the first check. It was this final triple-checked version that was administered. The original English version is presented in Appendix C in Supplementary Material.

The pre-experiment questionnaire consisted of several demographic questions, as well as a concern for reputation scale and a self-construal scale, to check whether participants across the three samples really differed on these dimensions.

To measure self-construal, we administered one of the subscales (self-interest vs. commitment to others) of the initial version of Vignoles et al. (2016) measure of self-construal, which has been validated in multiple languages across 16 countries, including Mandarin. The higher one's score on this scale, the more interdependent (collectivistic) one's self construal. When first examined, the alphas were very low for the three samples (Taiwan, α = 0.50; Pakistan, α = 0.48; The Netherlands, α = 0.52). Upon further examination, it became clear that the final two items (“I should be judged on my own merit” and “I am comfortable being singled out for praise and rewards”), were actually negatively correlated with the rest of the items in the scale. We suspect that these two items—the only two in the scale that were reverse-coded to measure interdependence/collectivism—were in fact measuring independence/individualism as a separate construct instead of merely the inverse of interdependence. We thus removed these items from our analyses. After this procedure, the alpha's for the difference samples (Taiwan, α = 0.72, Pakistan, α = 0.68, and The Netherlands, α = 0.65) all presented an acceptable reliability.

To measure participants' concern for reputation, we employed a modified version of de Cremer and Tyler (2005). Concern for Reputation scale (CfR). This scale has been validated in both English- and Italian-speaking populations to date (see Cavazza et al., 2014). To capture all types of reputation described earlier—honor, face, and dignity—this initial scale was expanded to nine items, three for each reputational construct. Participants were asked to indicate on a scale of 1 (not at all) to 5 (extremely) to what extent they felt these statements applied to them. The alphas were acceptable across the three samples: Taiwan = 0.73, Pakistan = 0.62, and The Netherlands = 0.73). Because there were so few items per dimension, and because reputation is only a part of the honor-face-dignity framework, we chose to treat this as a single measure of concern for reputation, although we included representations reflecting each cultural logic (see Leung and Cohen, 2011).

In the post-experiment questionnaire, we measured participants' emotional responses to the game and their behavioral intentions toward the two computer players who they still believed to be other human participants. To assess participants' emotional responses after the game, we used a modified version of the Discrete Emotions Questionnaire (DEQ; Harmon-Jones et al., 2016). This is a popular emotional evaluative tool that has been used in several different cultural contexts (see Megías et al., 2011; Yilmaz and Bekaroglu, 2020). Because we were only interested in a few specific emotions, and also wanted to preserve the engage-disengage paradigm put forth by Kitayama et al. (2006), we kept the DEQ's format and instructions, but only presented ten items divided into five two-item subscales: positive disengaging emotions (proud, confident), negative disengaging emotions (angry, mad), positive engaging emotions (happy, cheerful), negative engaging emotions (embarrassed, humiliated), and the general construct of respect (respected, ashamed).

Because our interest was in negative emotions resulting from trolling behaviors, we focused on the negative disengaging and engaging emotion examples only in our analyses. However, it is worth noting that the item “ashamed” from the respect construct correlated with both negative engaging emotions in Pakistan (embarrassed, *r* = 0.57; humiliated, *r* = 0.53), and the Netherlands (embarrassed, *r* = 0.32; humiliated, *r* = 0.59), while correlating with the “humiliated” item in Taiwan (*r* = 0.38). The “respected” item from the respect construct correlated with both positive approach emotions near equally in Taiwan (proud, *r* = 0.53; confident, *r* = 0.53), Pakistan (proud, *r* = 0.60; confident, *r* = 0.53), and the Netherlands (proud, *r* = 0.53; confident, *r* = 0.57). The items “angry” and “mad,” our disengaging emotions, correlated in all three samples (Taiwan, *r* = 0.89; Pakistan, *r* = 0.75; The Netherlands, *r* = 0.71), while the items “embarrassed” and “humiliated,” our engaging emotions, only correlated in the Pakistan (*r* = 0.63) and Dutch sample (*r* = 0.42). In Taiwan, the correlation was negligible (*r* = 0.11). We therefore ran our initial analyses with the two items combined, and then another analysis with them separate to determine which item was driving the effects in our earlier tests.

To assess participants' behavioral intentions, we used an adapted version of a scale used by Schaafsma and Williams (2012) when they aimed to assess aggressive behavioral intentions across cultural groups in the Netherlands—more specifically, contrasting honor- and dignity-valuing cultures. We reduced the scale to six items—two aggressive intentions (e.g., “hurt the other players”), two reparative intentions (“have a chat with the other players”), and two withdrawal intentions (e.g., “stay away from the other players”). During the translation process, we also shortened the scale from seven to five points, and worded the directions to “indicate … how much you want to” and had 1 = not at all, and 5 = extremely. The items representing withdrawal—“stay away from the other players” and “avoid the other players in real life”—were correlated in all three samples (Taiwan, *r* = 0.66; Pakistan, *r* = 0.61; The Netherlands, *r* = 0.29), although the correlation in the Dutch sample was weaker than in the other samples. The items representing aggression—“hurt the other players” and “swear at the other players”—also correlated in all three samples (Taiwan, *r* = 0.55; Pakistan, *r* = 0.73; The Netherlands, *r* = 0.66). The items representing relationship building or repairing—“have a chat with the other players” and “meet the other players”—correlated weakly, but still significantly, in Taiwan (*r* = 0.24), and strongly in Pakistan (*r* = 0.59), and the Netherlands (*r* = 0.61).

To verify whether the participants were negatively affected by our trolling manipulation, we concluded the post-experiment questionnaire with four manipulation check questions. These consist of an “I felt …” statement (liked, rejected, humiliated, ridiculed), followed by a scale from 1 (not at all) to 5 (extremely). Participants were instructed to “select the number that best represents the feelings you experienced during the game” for each statement. The items were acceptably reliable in all three samples (Taiwan, α = 0.78; Pakistan, α = 0.79; The Netherlands, α = 0.84).

Participants' messages were also coded as a behavioral measure. From 10 basic codes that were given to each message sent by our participants, we reduced it to three macro-codes: retaliation, reparation, and miscellaneous. The number of messages coded as either retaliation or reparation (attempts to repair the relationship) were used as our behavioral measure in our analyses. The full coding procedure is presented in Appendix A in Supplementary Material.

## Results

### Analytical Strategy

To examine the effects of the three independent variables on the various dependent variables (emotional reactions, behavioral intentions, and behavior during the game), we first ran a series of three-way MANCOVAs whereby nationality (Taiwanese, Pakistani, Dutch), trolling type (flaming, ostracism, control), and perpetrator group membership (ingroup, outgroup) were included as the between-subjects factors and either anger or embarrassment (emotions), aggression, reparation or withdrawal (intentions), or reparation and retaliation (behavior) as within-subjects factors. Means and standard deviations for each of these according to the between-subject factors listed are presented in Table 1. Gender and age were included as covariates in these analyses, but neither ever produced a significant effect—either on their own (Age: η^2^ < 0.001, *p* > 0.07, observed power < 0.09; Gender: η^2^ < 0.001, *p* > 0.76, observed power < 0.06) or in interactions (Age: η^2^ < 0.007, *p* > 0.10, observed power < 0.38; Gender: η^2^ < 0.001, *p* > 0.11, observed power < 0.36)—and so they were removed from final analyses. A correlation matrix of our between-subjects factors is presented in Table 2. Because neither the behavioral measures (retaliation and reparation), nor all of the behavioral intention measures (only aggression and withdrawal, not the intention for reparation) correlated significantly, we chose to run two MANOVAs—one with emotions (anger/embarrassment) as a within-subjects variable, and one with negative intentions (aggression/withdrawal)—and three ANOVAs with the intention to repair the relationship, behavioral retaliation, and behavioral reparation as their respective dependent variables.

**Table 1:** Descriptive statistics of within-subjects factors and dependent variables.

**Nationality**	**Trolling**	Group^*^	***N***	**Anger**			**Embarrassment**			**Aggression**			**Withdrawal**			Reparation^**^	
				***M***	***SD***	**CI**	***M***	***SD***	**CI**	***M***	***SD***	**CI**	***M***	***SD***	**CI**	***M***	***SD***
Taiwanese	None	In	25	1.46	1.04	[1.03, 1.89]	2.40	0.63	[2.14, 2.66]	1.22	0.76	[0.90, 1.54]	1.78	0.97	[1.38, 2.18]	2.65	0.83
		Out	23	1.20	0.49	[0.98, 1.41]	2.37	0.80	[2.02, 2.72]	1.07	0.23	[0.97, 1.16]	1.76	0.67	[1.47, 2.05]	2.82	0.91
	Ostracism	In	21	2.64	1.16	[2.11, 3.17]	3.21	1.04	[2.74, 3.69]	1.57	0.87	[1.18, 1.97]	2.45	1.30	[1.86, 3.05]	3.00	0.81
		Out	20	2.55	1.18	[2.00, 3.10]	3.38	1.06	[2.88, 3.87]	1.65	0.69	[1.33, 1.97]	2.90	1.12	[2.38, 3.42]	3.17	1.06
	Flaming	In	25	2.86	0.90	[2.49, 3.23]	3.48	0.90	[3.11, 3.85]	1.74	0.95	[1.35, 2.13]	3.40	0.91	[3.02, 3.78]	2.98	1.04
		Out	25	2.78	1.23	[2.27, 3.29]	3.56	0.65	[3.29, 3.83]	1.92	1.06	[1.48, 2.36]	2.98	0.99	[2.57, 3.39]	2.60	0.68
Pakistani	None	In	26	1.79	0.96	[1.40, 2.18]	1.52	0.66	[1.25, 1.78]	1.64	0.86	[1.29, 1.98]	1.87	1.03	[1.45, 2.28]	2.76	1.30
		Out	25	1.72	0.82	[1.38, 2.06]	1.30	0.46	[1.11, 1.49]	1.50	0.72	[1.20, 1.80]	2.02	0.96	[1.62, 2.42]	3.29	1.24
	Ostracism	In	24	2.77	1.22	[2.26, 3.28]	2.69	1.14	[2.21, 3.17]	1.83	1.03	[1.40, 2.27]	3.04	1.40	[2.45, 3.63]	2.93	1.18
		Out	23	2.52	1.29	[1.36, 2.32]	2.24	1.20	[1.72, 2.76]	2.20	1.28	[1.64, 2.75]	2.72	1.20	[2.20, 3.24]	3.02	1.15
	Flaming	In	25	1.84	1.15	[1.86, 2.75]	1.82	1.03	[1.40, 2.25]	1.62	0.78	[1.30, 1.94]	2.04	0.92	[1.66, 2.42]	2.60	0.88
		Out	26	2.31	1.11	[1.05, 1.57]	1.83	0.85	[1.48, 2.17]	1.89	1.03	[1.47, 2.30]	2.90	1.17	[2.43, 3.38]	3.30	0.89
Dutch	None	In	21	1.31	0.58	[1.14, 1.77]	1.86	0.71	[1.53, 2.18]	1.10	0.34	[0.94, 1.25]	1.50	0.61	[1.22, 1.78]	2.48	0.96
		Out	22	1.46	0.71	[1.77, 2.56]	1.96	1.05	[1.49, 2.42]	1.23	0.53	[0.99, 1.46]	1.77	0.97	[1.34, 2.20]	2.50	1.10
	Ostracism	In	24	2.17	0.94	[1.69, 2.54]	3.35	0.84	[3.00, 3.71]	1.67	0.82	[1.32, 2.01]	2.69	0.96	[2.28, 3.10]	2.68	1.03
		Out	22	2.11	0.96	[1.53, 2.24]	3.21	0.87	[2.82, 3.59]	1.46	0.58	[1.20, 1.71]	2.61	1.20	[2.08, 3.15]	2.35	1.10
	Flaming	In	22	1.87	0.80	[1.58, 2.55]	2.59	0.93	[2.18, 3.01]	1.52	0.63	[1.25, 1.80]	2.93	1.26	[2.38, 3.49]	2.07	1.05
		Out	24	2.06	1.15	[1.98, 2.19]	2.71	1.23	[2.19, 3.23]	1.56	0.71	[1.26, 1.86]	2.65	1.26	[2.11, 3.18]	2.36	1.01

* *Troll's group membership (in-group or out-group). CI, confidence intervals*.

** *Proportion of total messages sent*.

**Table 2:** Correlation matrix of all dependent variables.

	**Anger**	**Embarrassment**	**Aggression**	**Withdrawal**	**Reparation (I)**	**Retaliation**	**Reparation**
Anger	**1.00**						
Embarrassment	**0.56**	**1.00**					
Aggression	**0.46**	**0.22**	**1.00**				
Withdrawal	**0.48**	**0.43**	**0.45**	**1.00**			
Reparation (I)	**0.02**	−0.07	0.07	**−0.21**	**1.00**		
Retaliation	**0.16**	0.08	**0.18**	**0.19**	0.01	**1.00**	
Reparation	**0.11**	0.07	**0.14**	0.04	**0.25**	**−0.25**	**1.00**

*Reparation (I) refers to the behavioral intent to repair the relationship, while Reparation refers to the actual behavior of sending a message to repair/build the relationship. Bolded correlations are significant at the 0.05 level*.

Prior to conducting these analyses, we ran a two-way ANOVA with nationality, trolling type, and perpetrator group membership as the independent variables, to examine whether our manipulations had a discernible effect on how liked or rejected participants felt. We also conducted one-way ANOVAs with nationality as the independent variable to check whether or not our three cultural settings differed significantly in terms of our two cultural variables: self-construal and reputation. In addition, a *post-hoc* power analysis in G^*^Power (Faul et al., 2007, 2009) revealed that the study is sufficiently powered to successfully detect effects as small as *f* ^2^ = 0.02, or 2% of the total variance explained (a small effect).

### Preliminary Analyses

Our initial ANOVA revealed that both trolling type [*F*_(2,421)_ = 90.12, *p* < 0.001, η^2^ = 0.30] and nationality [*F*_(2,421)_ = 13.07, *p* < 0.001, η^2^ = 0.06] had significant effects on our manipulation questions (liked and rejected feelings), but that there was also a significant interaction between these two predictors, *F*_(4,421)_ = 7.13, *p* < 0.001, η^2^ = 0.07. Participants felt more rejected when flamed or ostracized (means ranged from 2.51 to 3.69) than when in a control condition (means ranged from 2.06 to 2.16), showing that our primary manipulation was successful across all samples tested. In Taiwan, participants felt the most rejected in the flaming condition (*M* = 3.69, *SD* = 0.73), while in Pakistan (*M* = 3.20, *SD* = 1.07), and The Netherlands (*M* = 3.36, *SD* = 0.83), participants felt the most rejected in the ostracism condition. The perpetrator's group membership did not significantly predict our manipulation check results, *F*_(1,421)_ = 0.01, *p* = 0.90, η^2^ < 0.001, observed power = 0.05.

An ANOVA also confirmed that there are significant differences between the countries in terms of both self-construal [*F*_(2,421)_ = 8.97, *p* < 0.001, η^2^ = 0.04] and concern for reputation, *F*_(2,422)_ = 48.91, *p* < 0.001, η^2^ = 0.19. For self-construal, a Tukey's honest significant difference test revealed that Taiwanese (*p* = 0.04, *d* = 0.31) and Pakistani participants (*p* < 0.001, *d* = 0.50) were significantly more interdependent than Dutch participants. There was no significant difference in this regard between the Taiwanese and Pakistani participants, *p* = 0.19, *d* = 0.20. For reputation, we found that Taiwanese participants were the most concerned about their reputation, followed by Pakistani participants, with the Dutch being the least concerned, all *p*s < 0.001, Taiwanese to Pakistani, *d* = 0.60; Taiwanese to Dutch, *d* = 1.20; Pakistani to Dutch, *d* = 0.57. These findings confirm the idea that people from face and honor settings see themselves as more interdependent and also tend to be more concerned about their reputation than those from dignity settings.

### Emotional Responses to Flaming and Ostracism

We expected that participants from a culture that values honor (Pakistan) would experience more anger when flamed by outgroup members than by ingroup members (Hypothesis 1a) and feel more embarrassed when ostracized by ingroup members than by outgroup members (Hypothesis 1b). For participants from a face-valuing culture (Taiwan), we anticipated that they would feel more embarrassment when flamed or ostracized by ingroup members than by outgroup members (Hypothesis 2a and 2b, respectively). However, we did not find three-way interactions between nationality, trolling type, and group membership when it comes to negative emotions, *F*_(4,405)_ = 0.12, *p* = 0.98, η^2^ = 0.001, observed power = 0.08.

**Figure 1:**
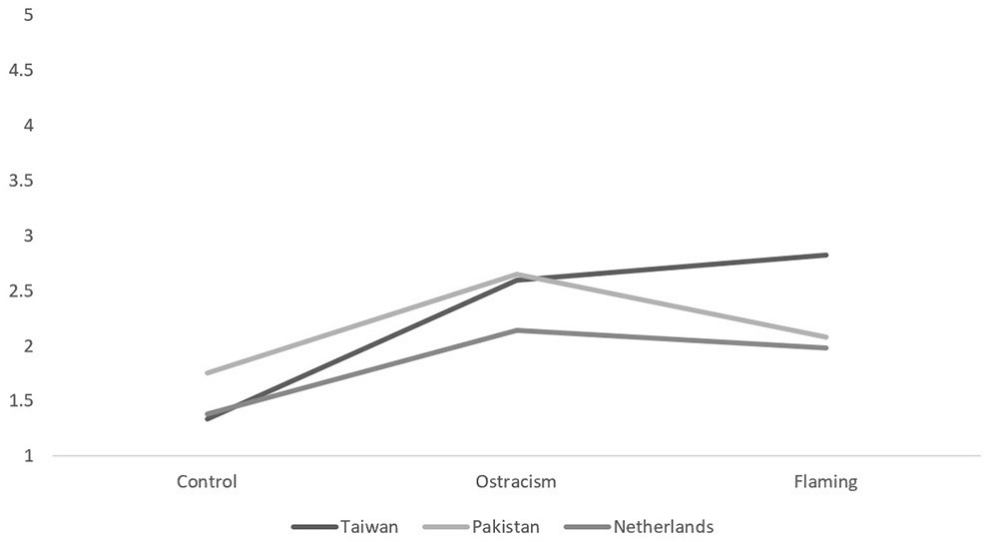
The between-subjects interaction between nationality and trolling type for anger.

**Figure 2:**
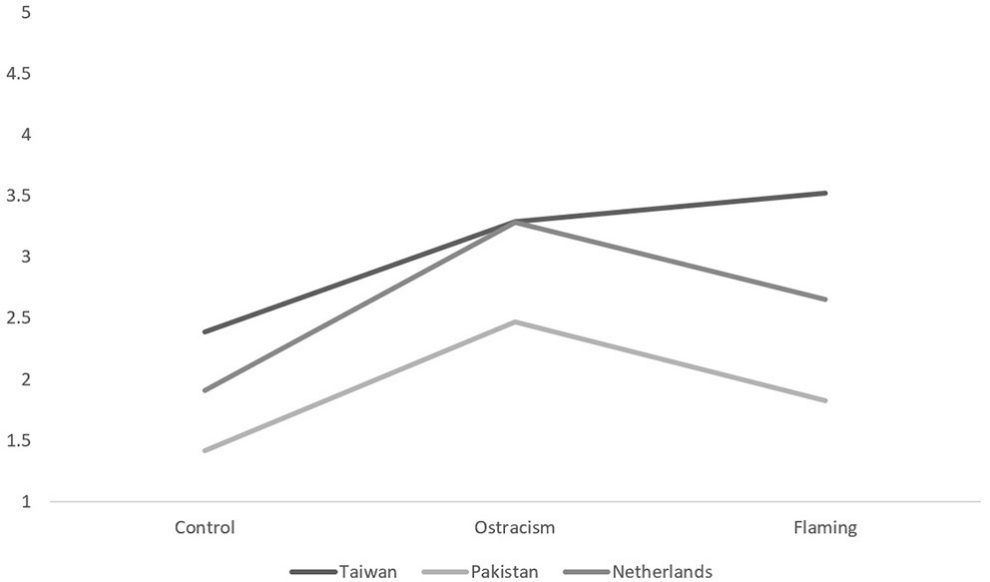
The between-subjects interaction between nationality and trolling type for embarrassment.

The analyses did reveal significant main effects of nationality, trolling type, and type of emotion (engaging or disengaging), and a significant interaction between nationality and type of emotions and nationality and trolling type (*F*s > 4.85, *p*s < 0.001). This last two-way interaction between nationality and trolling type is visualized in Figure 1 (anger) and Figure 2 (embarrassment). Simple effects for this interaction revealed that the difference between the flaming and ostracism conditions in Taiwan [*F*_(1,420)_ = 26.38, *p* < 0.001, η^2^ = 0.06], Pakistan [*F*_(1,420)_ = 25.97, *p* < 0.001, η^2^ = 0.06], and the Netherlands [*F*_(1,420)_ = 26.79, *p* < 0.001, η^2^ = 0.06] were all significant. It would thus appear that Pakistani participants experienced the most anger and embarrassment while ostracized, running counter to our expectation that flaming would produce primarily anger (H1a) and ostracism primarily embarrassment (H1b), while Taiwanese participants experienced the most anger *and* embarrassment while flamed, contrary to our supposition that they would experience primarily embarrassment across both trolling types (H2a and H2b).

This two-way interaction is given additional nuance, however, from a significant interaction between nationality, trolling type, and type of emotion, *F*_(4,405)_ = 3.39, *p* = 0.01, η^2^ = 0.03. The simple effects tests that we conducted to examine this revealed a significant two-way interaction between negative emotions and trolling for the Dutch sample only, *F*_(2,416)_ = 4.56, *p* = 0.01. A further inspection of this interaction revealed that they experienced significantly more embarrassment than anger in the control [*F*_(1,420)_ = 10.92, *p* = 0.001], ostracism [*F*_(1,420)_ = 55.56, *p* < 0.001], and flaming conditions [*F*_(1,420)_ = 19.37, *p* < 0.001].

Given that the two engaging negative emotion items (embarrassment and humiliation) were not correlated in the Taiwan sample, we conducted two additional ANOVAs with the same between-subjects variables as before, and the two items, “embarrassed” and “humiliated,” as dependent variables. These analyses revealed that in the Taiwan sample, the mean levels of embarrassment were similar in the trolling and non-trolling conditions [*F*_(2,414)_ = 1.94, *p* = 0.15], but there were significant differences in humiliation between the flaming and ostracism conditions, *F*_(1,414)_ = 83.46, *p* < 0.001. Flaming (*M* = 3.64, *SD* = 1.05) resulted in more humiliation than ostracism (*M* = 2.61, *SD* = 1.38), *F*_(1,414)_ = 23.06, *p* < 0.001, and these two trolling conditions resulted in more humiliation than the control condition [*F*_(1,414)_ = 83.46, *p* < 0.001]. Overall, these results would suggest that the effect we found earlier—the lack of difference between flaming and ostracism—is because of the embarrassment item, as we do find a difference between the two in terms of humiliation.

### Behavioral Intentions Toward Perpetrators of Flaming and Ostracism

In terms of behavioral intentions, we expected that our honor-valuing culture participants would express more aggressive intentions, particularly when flamed by outgroup members (Hypothesis 1a), but that they want to try to repair the relationship when ostracized, particularly by ingroup member (Hypothesis 1b). For our face-valuing culture participants, we anticipated that flaming (by ingroup members in particular) would lead them to withdraw, but that ostracism by ingroup members would lead to participants trying to restore their relationship with the perpetrator (Hypothesis 2b). It is important here to note that aggressive intention scores were low across all cultures; none of our sample could be considered truly “aggressive” in their responses.

**Figure 3:**
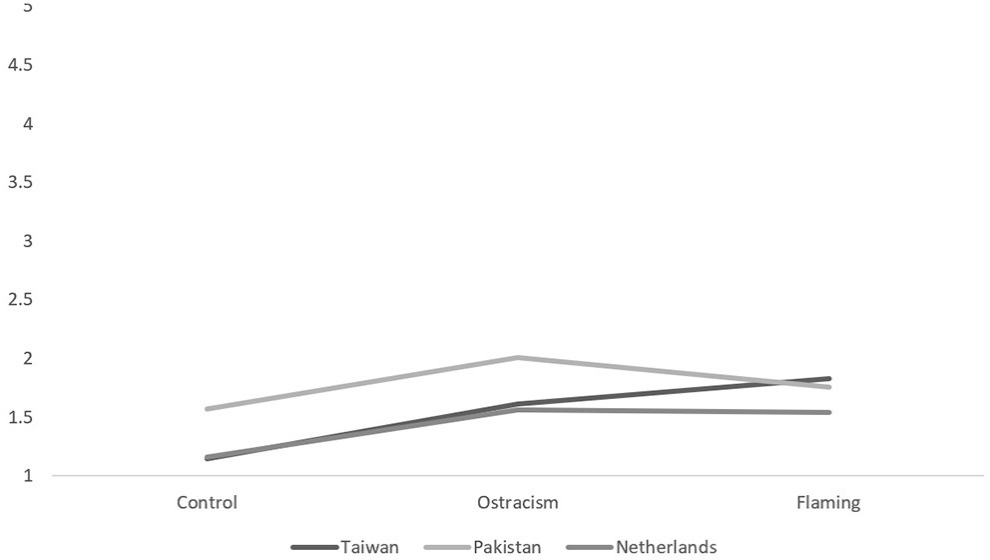
The between-subjects interaction effect between nationality and trolling type for aggressive intentions.

**Figure 4:**
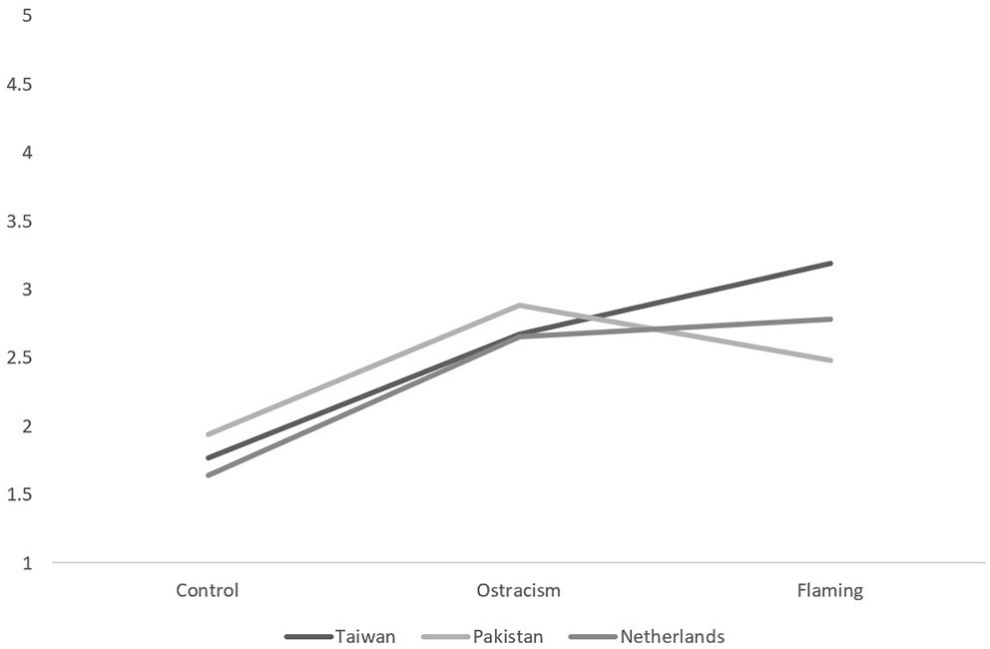
The between-subjects interaction between nationality and trolling type for withdrawal intentions.

For the negative behavioral intentions, our analysis revealed significant main effects of trolling type and negative intentions, as well as significant interactions between, nationality and negative intentions, trolling type and negative intentions, and another between nationality and trolling type (*F*s > 4.77, *p*s < 0.01). This last interaction is visualized in Figure 3 (aggression) and Figure 4 (withdrawal). Simple effects analyses revealed that the differences in negative behavioral intentions—both anger and withdrawal—between the flaming and ostracism conditions were significant in Taiwan [*F*_(1,420)_ = 14.74, *p* < 0.001, η^2^ = 0.03], Pakistan [*F*_(1,420)_ = 15.70, *p* < 0.001, η^2^ = 0.04], and the Netherlands, *F*_(1,420)_ = 15.07, *p* < 0.001, η^2^ = 0.03. Our Taiwanese participants thus wanted to aggress *and* withdraw the most when flamed, contradicting the idea that withdrawal would be expressed equally between trolling conditions (H2a and H2b). Nevertheless, it does partially support our prediction in H1a that Pakistani participants would feel heightened anger when flamed, although perpetrator group membership did not play a role in this experience.

However, our Pakistani participants were also involved in a within-between effects four-way interaction between nationality, trolling type, troll's group membership, and negative intentions, *F*_(4,405)_ = 3.21, *p* = 0.01, η^2^ = 0.04. This interaction was only significant in Pakistan [*F*_(2,416)_ = 4.65, *p* = 0.01; all other *p*s > 0.08]. Simple effects tests revealed that in the Pakistani sample, when participants were ostracized [*F*_(1,419)_ = 4.63, *p* = 0.03] by an in-group perpetrator, they intended to withdraw (*M* = 2.72, *SD* = 1.20) more than aggress (*M* = 2.20, *SD* = 1.28), but when faced with an out-group perpetrator, they intended to aggress (*M* = 3.04, *SD* = 1.40) more than withdraw (*M* = 1.83, *SD* = 1.03), which confirms what we predicted in H1b. No such difference was found for the Taiwan and Dutch participants.

For reparative intentions—we not only found no hypothesized three-way interaction [*F*_(4,405)_ = 1.34, *p* = 0.26], but we found no significant main effects (all *F*s < 2.02, all *p*s > 0.16) or lower-order interactions (all *F*s < 2.33, all *p*s > 0.10) at all. This would suggest that participants across all countries experienced equal desire (all means ranged from 2.06 to 3.17) to meet and befriend the computer players, irrespective of whether these players trolled them or not. That said, these means are quite low, suggesting that few participants had any desire to actually go and meet the other players or befriend them in person. This also goes against the idea as expressed in hypotheses 1b and 2b that members of face-valuing and honor-valuing cultures would want to repair the relationship with ingroup ostracizers, as neither our Taiwanese nor our Pakistani sample expressed any major desire to do so, irrespective of trolling condition or perpetrator group membership.

### Behavioral Responses to Flaming and Ostracism

Because the actual behavioral data—the messages participants sent during the game—consisted of count data, they could not be analyzed using the same techniques as the emotional responses and behavioral intentions. Since our interest was in relative usage (e.g., do Pakistani participants more often retaliate or try to repair the relationship?), we first calculated the proportion of total messages used to either retaliate or repair (in the case of no messages sent, a 0 was entered manually to signify that none of the messages sent pertained to retaliation or reparation) according to our coding scheme (see Table 1 and Appendix A in Supplementary Material). We then calculated the descriptive statistics for these proportions by nationality, trolling type, and troll's group membership, which are presented in Table 3.

**Table 3:** Descriptive statistics of participants' behavioral responses to trolling.

**Nationality**	**Trolling**	Group^*^	***N***	Retaliation^**^		Reparation^**^	
				***M***	***SD***	***M***	***SD***
Taiwanese	None	In	25	0.01	0.04	0.20	0.26
		Out	23	0.00	0.00	0.22	0.29
	Ostracism	In	21	0.08	0.17	0.52	0.28
		Out	20	0.02	0.06	0.53	0.24
	Flaming	In	25	0.33	0.25	0.23	0.25
		Out	25	0.38	0.28	0.25	0.24
Pakistani	None	In	26	0.06	0.18	0.40	0.36
		Out	25	0.03	0.10	0.34	0.41
	Ostracism	In	24	0.14	0.23	0.70	0.26
		Out	23	0.12	0.22	0.55	0.36
	Flaming	In	25	0.13	0.27	0.38	0.38
		Out	26	0.14	0.28	0.34	0.39
Dutch	None	In	21	0.09	0.21	0.18	0.23
		Out	22	0.03	0.14	0.33	0.35
	Ostracism	In	24	0.06	0.16	0.39	0.31
		Out	22	0.06	0.16	0.35	0.30
	Flaming	In	22	0.26	0.28	0.25	0.26
		Out	24	0.19	0.25	0.26	0.30

* *Troll's group membership (in-group or out-group)*.

** *Proportion of total messages*.

**Figure 5:**
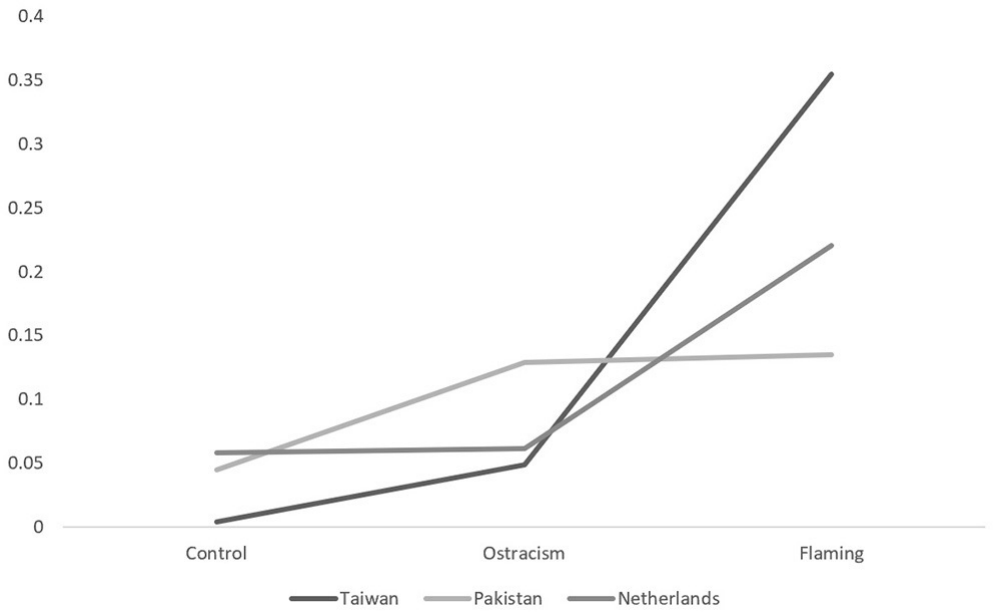
The interaction between nationality and trolling type for retaliation.

For behavioral aggression (retaliation), we did not find the three-way interaction we anticipated, *F*_(4,405)_ = 0.52, *p* = 0.72, η^2^ = 0.005, observed power = 0.18. We did, however, find a significant main effect of type of trolling [*F*_(2,405)_ = 39.18, *p* < 0.001, η^2^ = 0.16], as well as a significant interaction between nationality and trolling type, *F*_(4,405)_ = 8.31, *p* < 0.001, η^2^ = 0.08. No other interactions were significant (all Fs < 0.52, *p*s > 0.72), nor was there an effect of perpetrator group membership, F_(1,405)_ = 0.04, *p* = 0.33. The significant interaction between nationality and trolling type is visualized in Figure 5. Follow-up analyses for revealed that in Pakistan, participants responded with aggression significantly more when flamed or ostracized than when in the control condition [*F*_(1,414)_ = 6.35, *p* = 0.01], but that there was no significant difference between the flaming and ostracism condition, *p* = 0.92. This partially supports our expectation that participants from honor-valuing cultures (Pakistan) would be more likely to react aggressively following flaming from outgroup members. We found that Taiwanese participants, however, were more likely to aggress when trolled (flamed or ostracized) compared to the control condition [*F*_(1,414)_ = 29.63, *p* < 0.001] and also more in the flaming than in the ostracism condition, *F*_(1,414)_ = 53.22, *p* < 0.001. We found the same pattern among our Dutch participants, who also reacted with aggression more when trolled than when in a control condition [*F*_(1,414)_ = 4.91, *p* = 0.03], and more when flamed than when ostracized, *F*_(1,414)_ = 14.33, *p* < 0.001. This again goes against the idea that face-valuing culture members avoid aggression due to rejection avoidance tendencies (e.g., Hashimoto and Yamagishi, 2013, 2016), as our Taiwanese participants retaliated in both our ostracism and flaming conditions.

In terms of reparation, we expected that honor-valuing and face-valuing participants would try to repair the relationship, particularly when ostracized by ingroup members. We did not find, however, the hypothesized three-way interaction [*F*_(4,405)_ = 0.38, *p* = 0.82, η^2^ = 0.003, observed power = 0.14], nor did we find any lower-order interactions (all *F*s < 1.69, all *p*s > 0.19). Instead, we found two significant main effects: one of nationality [*F*_(2,405)_ = 10.24, *p* < 0.001, η^2^ = 0.05], and the other of trolling type, *F*_(2,405)_ = 23.90, *p* < 0.001, η^2^ = 0.11. A series of simple contrasts revealed that while Pakistani participants sent proportionately more reparation messages than both the Taiwanese [*F*_(1,414)_ = 13.00, *p* < 0.001] and Dutch [*F*_(1,414)_ = 16.97, *p* < 0.001] participants, there was no significant difference between the amount of reparation expressed by the Taiwanese and the Dutch, *p* = 0.59. Another series of simple contrasts showed that participants in the ostracism condition expressed proportionately more reparation than participants in the flaming [*F*_(1,414)_ = 36.89, *p* < 0.001] and control [*F*_(1,414)_ = 35.59, *p* < 0.001] conditions, and that there was no significant difference in terms of reparation between these latter two, *p* = 0.87. Thus, we cannot confirm that honor- and face-valuing culture members tend to engage in repairing the relationship when flamed (honor) or ostracized (honor and face) by ingroup members, as we found no interactions between nationality/culture, trolling type, or perpetrator group membership.

## Discussion

The aim of this study was to examine how people respond to two different types of trolling—flaming and ostracism—and whether this varies as a function of their cultural background and the perpetrator's group membership—ingroup or outgroup. Based upon previous theorizing and empirical work on honor and its connections with aggression, we expected that participants from honor-valuing cultural contexts would react with anger and aggression to flaming, particularly in the case of outgroup perpetrators, but would feel embarrassed and try to repair the relationship when ostracized, particularly when ostracized by ingroup members. We also expected that our participants from a face-valuing cultural context would generally feel embarrassed and try to withdraw and avoid conflict when faced with flaming, but would try to repair the relationship with ingroup ostracizers.

Our results provided mixed support for the idea that honor-valuing culture members should respond with anger and aggression when flamed by outgroup members (**H1a**). For example, although we did find that flaming resulted in anger among our Pakistani participants, it actually produced less anger than ostracism and also did not vary as a function of perpetrator group membership. After being flamed, Pakistani participants expressed the desire to withdraw from instead of aggress the perpetrator, particularly when the perpetrator was an ingroup member. During the flaming, Pakistani participants did react with aggression, but no more so than if they were being ostracized, and irrespective of the perpetrator's group membership. In terms of their reactions to ostracism, we found that they did generally withdraw emotionally and experience embarrassment when faced with ostracism, also irrespective of the perpetrators group membership, although no more than if they were faced with flaming (**H1b**). In terms of the emotional response to ostracism, there is some evidence in the literature to suggest that, although ostracism in particular is an extremely noxious experience for more collectivistic culture members—which we have confirmed is the case, as honor-valuing culture members did experience negative emotions after being ostracized—they may also recover from these experiences faster than their more individualistic counterparts, depending on how they respond in the moment (Pfundmair et al., 2014; Yaakobi and Williams, 2016b). Future research could give a longer delay between the ostracism and flaming experiences to see if there are differences in terms of how long this emotional reaction lasts in different cultural contexts.

After being ostracized, participants expressed the desire to aggress outgroup perpetrators and withdraw from in-group perpetrators though, which was consistent with our predictions. Pakistani participants were also the most likely to engage in reparation, as were participants in ostracism conditions, but there was no effect of perpetrator group membership. This final result (high rates of reparation) is likely due to the importance of reparation after conflict in Pakistani culture (Anjum et al., 2017). Although research would suggest that an action is preferred over words (Anjum et al., 2018), words are all that the present experimental paradigm allowed, and so it was the medium of reparation our Pakistani participants used.

Taken together, these findings go against the idea presented in literature that the presence of an obvious insult is what triggers aggression in honor-valuing culture members (e.g., Harinck et al., 2013), as reactions from our Pakistani participants to ostracism and flaming only differed slightly. Whether flamed or ostracized, Pakistani participants expressed anger and were equally likely to retaliate, despite the fact that flaming consists of direct insults (O'Sullivan and Flanagin, 2003), while ostracism's insult is implicit and left open to interpretation (Williams, 2009). This also appears to go against Pfundmair et al. (2015) findings that aggressive intentions follow ostracism in more collectivistic cultures. Our results would suggest that flaming and ostracism are equally threatening when it comes to a loss of honor; both are interpreted as being insulting and requiring of defense.

Our null findings when it came to perpetrator group membership (ingroup or outgroup) in the flaming conditions may have to do with, at least in Pakistan's case, our Pakistani participants' relationships with other Pakistani University students (the ingroup) and Afghani people (the outgroup). Research has shown that when it comes to intergroup communication and conflict among Pakistani people, a key mechanism behind the hostility, emotionally and intentionally, is group relative deprivation (Obaidi et al., 2019). In essence, if the victim feels underprivileged financially or socially compared to the other group, this will trigger a more extreme reaction emotionally and in terms of behavioral intentions. Although Pakistani participants did sometimes react with anger and hostile intentions, means were very low across all reactions, and withdrawal was a much more popular option overall; this could suggest that our Pakistani participants felt relatively equal to both other students and to Afghani migrants. It could also be that there are fundamental differences between cultural values' application with strong and weak ties, as discussed earlier in our implicit research question. Further research could confirm or deny this possibility by performing a replication that included socio-economic data, something that was not collected in the present study, or by directly manipulating the strength of a troll's tie to the victim/participant.

Another possible explanation for these results could be due to the way that honor is conceived in different cultural contexts. Honor is typically shared by the wider social group (Leung and Cohen, 2011), and unprovoked insults and willfully ignoring people are both likely in violation of local honor codes (e.g., Cohen et al., 1996; Anjum et al., 2019). Participants may have felt like they needed to defend the honor of their culture and homeland—thus consequently their own honor—by correcting this perceived misrepresentation of what is allowable in their country (e.g., Rodriguez Mosquera et al., 2008; Anjum et al., 2019). Though outgroup perpetrators do not share a nationality with the participant in the present experiment, they do reside in the same city, giving participants a reason to want them to act in accordance with local norms and properly represent their ingroup. From this perspective, it is not the presence or absence of an overt insult that creates retaliation, but rather the transgression of norms, rendering unwarranted flaming and ostracism equally reprehensible. This would require further in-depth research in other honor-valuing cultural contexts to confirm or deny, but the present study does make the validity of overt insult as a mechanism for reactive aggression uncertain.

Our finding that ostracism and flaming are both considered equally offensive in the Pakistani context is, however, in line with research on negative self-conscious emotions (e.g., Allpress et al., 2014; Prati and Giner-Sorolla, 2017; Giner-Sorolla, 2019), and would suggest that both flaming and ostracism present a threat to a person's social identity (Chen et al., 2020; Dasborough et al., 2020). This also suggests that physical and verbal aggression is used to rebuff minor social infractions in honor-valuing cultural contexts primarily when face-to-face (Harinck et al., 2013; Severance et al., 2013). If this is indeed the case, our results may mean that the online context somehow levels the playing field and makes overt and covert aggression equally hurtful. This would, however, require further research to confirm or deny, and if it is indeed the case, the exact mechanism behind this effect remains unclear.

In terms of our expectation that participants from a face-valuing culture would be embarrassed and withdraw when faced with ingroup flaming (**H2a**), we again found only partial support. Emotionally, our Taiwanese did indeed experience embarrassment when flamed, which is in line without our expectations and also self-conscious emotion research (Allpress et al., 2014; Dasborough et al., 2020). However, there was no evidence to suggest that they intend to withdraw after being flamed any more than they do after being ostracized, and instead of trying to repair the relationship with the perpetrator, Taiwanese participants were likely to retaliate against their aggressor, irrespective of that person's group membership. We also expected our face-valuing culture participants to feel embarrassed and want to try to repair the relationship with ingroup ostracizers (**H2b**), but this was also not fully supported by our results. Although our Taiwanese participants did feel embarrassed when ostracized, they seemed to have mixed or uncertain intentions toward the perpetrator after the fact, and did not appear to retaliate any more than they engaged in reparation during the game. Once more, we found no effect of perpetrator group membership.

These results are surprising—especially the unexpectedly high retaliation and aggression rates among Taiwanese participants—as they appear to contradict the vast majority of the literature on face-valuing cultures, although they are in line with studies that focus on honor in traditionally face-valuing cultural contexts (e.g., Anjum et al., 2019). Cross-cultural studies often paint face-valuing cultures as being bent on rejection avoidance (e.g., Hashimoto and Yamagishi, 2013, 2016). Their bottom line is fitting in and avoiding stirring up conflict, as aggressive conduct is considered shameful and is likely to result in a loss of face (Leung and Cohen, 2011) for the person and their close others (see Markus and Kitayama, 1991). However, the context in which this study takes place—the internet—could provide a theoretical explanation for our findings in Taiwan. Just as our participants have a set of norms to which they generally adhere in their daily life—what we call their culture—the internet itself also has its own social norms (see Phillips, 2016). Our participants are taking their own cultural values into a unique culture in and of itself when they go online. One thing that has been repeatedly demonstrated about the internet's global culture is that in it, trolling in all its forms is exceedingly common (see Phillips, 2016; Cook et al., 2018). By retaliating against flaming, although our participants are contravening the norms of their own culture, they are actually fitting in to the internet's culture.

Another possible explanation of our results could be the anonymity of the internet and the perceived closeness of our Taiwanese participants to their fellow Taiwanese students. Online, no one can be sure of who you are (see Postmes et al., 1998), which means that no one can associate what you do with any of your close others. While in an offline situation, a person from a face-valuing cultural context would be risking a loss of face for themselves and their ingroup (Leung and Cohen, 2011; Hashimoto and Yamagishi, 2013, 2016), anonymity can prevent that loss of face entirely, as no one could connect their aggression to their group. It could be that the online context simply gave our participants the freedom to give knee-jerk reactions instead of having to consider any face-related consequences. It is also possible that even in face-valuing cultures, the direct insults make honor concerns more salient in the present study than face concerns, hence the results being more in line with Anjum et al. (2019) study as opposed to the work of Hashimoto and Yamagishi (2013, 2016).

Finally, we must consider our results in the dignity-valuing context. Though we had no specific hypotheses regarding our Dutch participants, they were intended to act as a comparison group, a representation of dignity-valuing (Leung and Cohen, 2011), independent (Markus and Kitayama, 1991) culture. Again, our results here were not in line with previous literature. Instead of feeling primarily anger (Rodriguez Mosquera et al., 2008), Dutch participants felt mostly embarrassment when trolled, and there were no distinctions between ostracism and flaming. They also retaliated the least of our three samples, irrespective of trolling type, when extant literature suggests that a more independent self-construal usually leads to retaliation (see Ma and Bellmore, 2016). From a theoretical standpoint, this is difficult to explain. It is possible that the anonymity of the internet removed the urgency from the situation; it is much harder to ignore people insulting a person to their face as opposed to from behind a screen (see Kyom, 2016). Future studies could explore this idea by explicitly measuring the importance of the medium to participants' lives and communication. There is also the fact that individualism is associated with less social interdependence and a more avoidant way of life, which is associated with less distress in general after being ostracized (Yaakobi and Williams, 2016b). This effect, however, has never been explored in terms of reactions to flaming. It could be that individualistic culture members are simply less distressed by insult and ostracism overall, experiencing light embarrassment instead of anger or distress. This would take further research to test, however.

There is, however, an additional question that this study brings up that could merit further exploration: the difference between an ingroup/outgroup effect and a majority/minority effect. In previous literature, majority vs. minority group situations have been used to simulate ingroups and outgroups, achieving the same effects experimentally (see Schaafsma and Williams, 2012); however, minority and majority group relations have their own separate literature as well (e.g., Hewstone et al., 1993; Jackson, 2002; Dixon et al., 2017; Gedeon et al., 2020). Much of this literature focuses on racial majorities and minorities (e.g., Rattan and Ambady, 2013; Benner and Wang, 2017; Gedeon et al., 2020), which are only a major factor in one of our ingroup/outgroup pairings (Dutch and Moroccans). In the majority/minority literature that does not specifically examine race, majorities and minorities seem to function largely the same way ingroups and outgroups do: differences between the two groups are exaggerated and similarities minimized (Hewstone et al., 1993). Thus, with the exception of our Dutch sample—in which there were no group effects to speak of anyway—majority vs. minority effects are essentially analogous to ingroup vs. outgroup effects. This not the case in every study that uses this kind of experimental design, though. Future work should be careful to take into account any racial data, while also assessing national identification (Jackson, 2002) and previous intergroup contact (Stathi and Crisp, 2008; Schmid et al., 2017) in addition to the cultural variables we used in order to ensure that they are capturing the construct they intend to, be that a majority vs. minority effect, or an ingroup vs. outgroup effect.

### Limitations

Despite our intriguing results, this study is not without its limitations. First among these is our sample of University students. Although they fit into the age range of some of the heaviest internet users and trolls (see Cook et al., 2018), using University students for experiments comes with its own risks. Across all countries, we had a minimum of 50% successful guess rate when we asked participants what they thought the study was about, despite our cover story. This is likely to be because all three participating universities had some form of computer science program in which artificial intelligence and chat-bots were featured. Although every effort was made to make it look like real people were playing, the salience of chat-bots among the student populations tested cannot be denied. Although research has found that, even when participants are aware of a perpetrator being a machine, it does not change the negative effects of online hostility (Zadro et al., 2004), the ecological validity would have been boosted significantly if fewer participants guessed the study's true purpose. There may also be a question of power, as the effect sizes in the present study were notably small; future studies should aim to have even more participants to detect even smaller effects than we were able to in the present work. We are also unsure of how salient our ingroup/outgroup manipulation was, and this could have also contributed to the lack of results when it came to that variable. Beyond this, our sample was relatively small for a cross-cultural study, and would have been more powerful with additional participants, preferably from a variety of universities within the countries in question to compensate for participants' potential familiarity with AI agents like chat-bots. Future studies should actively take media experience and technological familiarity into account, even when the primary interest is in cultural effects.

One final important limitation of the present study is the potential confound inherent to the study design when it comes to disentangling ostracism and flaming. Because we used pre-programmed confederates for consistency, natural responses to participants' messages or inquiries were not possible. This means that in both the control conditions and flaming conditions, participants did experience a form of ostracism (their messages receiving no response), albeit not as total as the one they experienced in the actual ostracism conditions. In addition, although it was intended to serve as a passive bystander—something that is seen quite regularly in online contexts (see Cook et al., 2018)—in the flaming condition, the “bystander” confederate also served as a co-victim, as the troll confederate periodically insulted them as well, while in the ostracism condition, they served as co-troll, as they did not address the participant either. Although it is evident that the key element of verbal insult was unique to the flaming conditions, and keeping the ball away from the participant was unique to the ostracism conditions, some forms of ostracism did likely bleed through all conditions. Thus, while we still found significant differences between the types of trolling in terms of the emotional response, intentions, and behavioral response, future studies performing comparisons of this kind should be extremely careful to ensure that they are fully separate. If they intend to use pre-programmed confederates as we did, advances in natural language processing might make this easier, while also boosting the ecological validity. Further branching scripts with human confederates may also help in this endeavor.

### Conclusions and Future Directions

So which is worse: ostracism or flaming? Our results do not offer a firm conclusion, but rather a resounding “it depends.” Emotionally, it would seem that flaming is a more intense experience for people from face-valuing cultural contexts, while ostracism is more intense for people from honor- and dignity-valuing cultural contexts. If “worse” is defined as producing more aggression, then flaming would be worse for face-valuing people, while ostracism would be worse for honor-valuing people. Dignity-valuing people produced so little aggression and retaliation when faced with either type of trolling that the two seem about even. Still, despite not giving a concrete answer to the question of which is worse, overt or covert aggression, the present study has advanced our understanding of both types of aggression in the online context in several ways. While Zadro et al. (2004) were among the first to compare ostracism and verbal aggression (flaming), our results expand upon their findings by looking at multiple indicators beyond the traditional effects (senses of belonging, control, self-esteem, and a meaningful existence) of ostracism. While earlier studies confirmed that responses to ostracism differ between cultural groups (e.g., Garris et al., 2011; Uskul and Over, 2014), the present study revealed that this is not just true of behavioral responses, but also emotional and intentional responses to not just ostracism, but also flaming, which has only limited cross-cultural studies in its extant literature (e.g., de Seta, 2013).

The study also joins the growing scholarship on online aggression, and opens up the question of how much influence the medium has on responses to both overt and covert aggression. In our dignity-valuing sample, for instance, flaming and ostracism were near equal in their effects: is this because of some mechanism related to being online, or something else? Extant literature frequently posits that dignity-valuing people are also the most provocative and retaliatory in their responses to aggression (e.g., Ma and Bellmore, 2016), but they were the least aggressive of all of our samples. Future studies should explore this further, manipulating not only the subtlety of the aggression, but also the medium, in order to isolate these sorts of effects. This type of study should also be conducted again in several cultural contexts, as there appear to be effects in non-honor-valuing cultures that are as of yet uncovered by existing theory (e.g., Dutch participants experiencing high levels of embarrassment) that could be specific to our samples. There is still much work to be done, and great opportunities for CMC, aggression, and cultural scholars to collaborate and explore this newest arena of hostility and intercultural communication.

## Data Availability Statement

The raw data supporting the conclusions of this article will be made available by the authors, without undue reservation.

## Ethics Statement

The studies involving human participants were reviewed and approved by Research Ethics and Data Management Committee (Tilburg School of Humanities and Digital Sciences). The patients/participants provided their written informed consent to participate in this study.

## Author Contributions

CC was in charge of conceptualizing the study, designing the study, coordinating the data collection across the three countries, performing all the analyses, and writing the full article. JS and MA assisted with the study design, devising an analytical strategy, and the editing of the written piece. SS and J-HL spearheaded the data collection in their respective countries and assisted with editing the final version of the article. HN was the data collection coordinator for the Netherlands, and also assisted with analyses. All authors contributed to the article and approved the submitted version.

## Conflict of Interest

The authors declare that the research was conducted in the absence of any commercial or financial relationships that could be construed as a potential conflict of interest.
