# Supplementary material for: Trolls Without Borders: A Cross-Cultural Examination of Victim Reactions to Verbal and Silent Aggression Online
Source: Front Psychol. 2021 Jun 1;12:549955. doi: 10.3389/fpsyg.2021.549955 (PMC8203910; doi:10.3389/fpsyg.2021.549955)
Supplement: Supplementary file 1 [file Data_Sheet_1.docx]

# Appendices

## Appendix A

In addition to their emotions and behavioural intentions, we were also interested in participants’ actual behaviour during the game. For this, we saved each individual chat message sent by the participant and coded them according to the schema presented in Table A (see supplementary files).

Two research assistants from each country who were native or near-native speakers of the language in question – either Mandarin, English, or Dutch – gave each individual message sent by our participants one of the codes listed in Table A, knowing the experimental condition. Each two-person team would meet via Skype with the primary investigator at four points during the coding: after 10 participants’ messages were coded, then 30 participants’, then 75 participants’, and a final time when all participants’ messages were coded. During these meetings, the coders and primary investigator would discuss messages that were coded differently by the coders and decide upon a final code together. Interrater reliability for this final coding of the last 70+ items was as follows: 81.9% in Taiwan, 87.8% in Pakistan, and 77.3% in the Netherlands.

From there, each code was sorted into one of three macro-categories: retaliation, reparation, and miscellaneous. Codes 1, 4, and 8 – rage, argumentation, and sarcasm and cynicism, respectively – fell under the retaliation category, as all of these possess either an element of disengaging emotions (rage and sarcasm) or conflict initiation. Codes 2, 3, 5, 7, and 8 – support-seeking, negotiation, rapport-building, sadness, and playfulness, respectively – fell under the reparation category, as all of these possess either an engaging emotion (sadness and playfulness) or engage positively with the other players. Codes 6 and 10 – neutrality and other – fell under the miscellaneous category, as they contained no particular emotional content, nor did they engage the other players in any way. It is the two of these three macro-codes – retaliation and reparation – that were used in our analyses.

## Appendix B

Welcome Message upon Entering Cyberball (English version)

Welcome to Cyberball! During this game, you will be passing a virtual ball to two other players by clicking on their avatars. To chat with them, click the chat box under your avatar, type, and hit enter or click ‘send’ to send. Your goal is to visualize the game while you play - imagine what the other players look like, where you are playing, the weather, etc.

Don't forget to introduce yourself to the other players too!

Scripts per Condition (English versions)

- General notes
  - For all conditions in the Netherlands, Player 3 gives the following introduction after 2 throws: “Hello, I’m Jeroen. I’m from Amsterdam, but study in Tilburg. I enjoy listening to music.”
  - For all conditions in Pakistan, Player 3 gives the following introduction after 2 throws: “Hello, I’m Ibrahim. I’m from Istanbul, but study in Lahore. I enjoy listening to music.”
  - For all conditions in Taiwan, Player 3 gives the following introduction after 2 throws: “Hello, I’m Jiawei. I’m from Tainan, but study in Taipei. I enjoy listening to music.”
- C1 = In-group Control
  - **Dutch:** Player 1 (computer) says the following script after the first ball toss – “Hi! My name is Thijs. I grew up in here in Tilburg. I’m a big fan of football!” The game then proceeds normally with random ball tosses and no further speech.
  - **Taiwanese:** Player 1 (computer) says the following script after the first ball toss – “Hi! My name is Guanlin. I grew up here in Taipei. I’m a big fan of soccer!” The game then proceeds normally with random ball tosses and no further speech.
  - **Pakistani:** Player 1 (computer) says the following script after the first ball toss – “Hi! My name is Ahmed. I grew up here in Lahore. I’m a big fan of football!” The game then proceeds normally with random ball tosses and no further speech.
- C2 = In-group Flaming
  - **Dutch:** Player 1 (computer) says the following script after the first ball toss – “Hi! My name is Thijs. I grew up in here in Tilburg. I’m a big fan of football!” Then, every 5 throws, Player 1 says one of the following items:
    - You’re so trashy I can smell you from here.
    - Do you not know how to use a computer? Loser.
    - I thought you had to be at least 18 to play this game? You play like a child.
    - You play like a girl.
    - I didn’t know you could be so bad at such an easy game.
  - **Taiwanese:** Player 1 (computer) says the following script after the first ball toss – “Hi! My name is Guanlin. I grew up here in Taipei. I’m a big fan of soccer!” Then, every 5 throws, Player 1 says one of the above items.
  - **Pakistani:** Player 1 (computer) says the following script after the first ball toss – “Hi! My name is Ahmed. I grew up here in Lahore. I’m a big fan of football!” Then, every 5 throws, Player 1 says one of the above items.
- C3 = In-group Ostracism
  - **Dutch:** Player 1 (computer) says the following script after the first ball toss – “Hi! My name is Thijs. I grew up in here in Tilburg. I’m a big fan of football!” The game then proceeds with neither Players 1 nor 3 (computers) passing to Player 2 (participant) for the duration of the game.
  - **Taiwanese:** Player 1 (computer) says the following script after the first ball toss – “Hi! My name is Guanlin. I grew up here in Taipei. I’m a big fan of soccer!” The game then proceeds with neither Players 1 nor 3 (computers) passing to Player 2 (participant) for the duration of the game.
  - **Pakistani:** Player 1 (computer) says the following script after the first ball toss – “Hi! My name is Ahmed. I grew up here in Lahore. I’m a big fan of football!” The game then proceeds with neither Players 1 nor 3 (computers) passing to Player 2 (participant) for the duration of the game.
- C4 = Out-group Control
  - **Dutch:** Player 1 (computer) says the following script after the first ball toss – “Hi! My name is Mohammed. I just moved here from Morocco. I’m a big fan of football!” The game then proceeds normally with random ball tosses and no further speech.
  - **Taiwanese:** Player 1 (computer) says the following script after the first ball toss – “Hi! My name is Danilo. I just moved here from the Philippines for work. I’m a big fan of soccer!” The game then proceeds normally with random ball tosses and no further speech.
  - **Pakistani:** Player 1 (computer) says the following script after the first ball toss – “Hi! My name is GulShar. I just moved here from Afghanistan. I’m a big fan of football!” The game then proceeds normally with random ball tosses and no further speech.
- C5 = Out-group Flaming
  - **Dutch:** Player 1 (computer) says the following script after the first ball toss – “Hi! My name is Mohammed. I just moved here from Morocco. I’m a big fan of football!” Then, every 5 throws, Player 1 says one of the following items:
    - You’re so trashy I can smell you from here.
    - Do you not know how to use a computer? Loser.
    - I thought you had to be at least 18 to play this game? You play like a child.
    - You play like a girl.
    - I didn’t know you could be so bad at such an easy game.
  - **Taiwanese:** Player 1 (computer) says the following script after the first ball toss – “Hi! My name is Danilo. I just moved here from the Philippines for work. I’m a big fan of soccer!” Then, every 5 throws, Player 1 says one of the above items.
  - **Pakistani:** Player 1 (computer) says the following script after the first ball toss – “Hi! My name is GulShar. I just moved here from Afghanistan. I’m a big fan of football!” Then, every 5 throws, Player 1 says one of the above items.
- C6 = Out-group Ostracism
  - **Dutch:** Player 1 (computer) says the following script after the first ball toss – “Hi! My name is Mohammed. I just moved here from Morocco. I’m a big fan of football!” The game then proceeds with neither Players 1 nor 3 (computers) passing to Player 2 (participant) for the duration of the game.
  - **Taiwanese:** Player 1 (computer) says the following script after the first ball toss – “Hi! My name is Danilo. I just moved here from the Philippines for work. I’m a big fan of soccer!” The game then proceeds with neither Players 1 nor 3 (computers) passing to Player 2 (participant) for the duration of the game.
  - **Pakistani:** Player 1 (computer) says the following script after the first ball toss – “Hi! My name is GulShar. I just moved here from Afghanistan. I’m a big fan of football!” The game then proceeds with neither Players 1 nor 3 (computers) passing to Player 2 (participant) for the duration of the game.

## Appendix C

**Pre-Test Questionnaire**

**Please answer the following questions either by filling in the blanks or circling the appropriate response provided.**

1. What is your age? _________________________________________
2. What is your gender? Male Female Other
3. What is your nationality?
   1. Taiwanese
   2. Pakistani
   3. Dutch
   4. Other _____________________________________________
4. What is your native language?
   1. Mandarin
   2. Taiwanese
   3. Urdu
   4. Hindi
   5. English
   6. Dutch
   7. Other _____________________________________________
5. What is your highest completed level of education?
   1. Primary school
   2. Middle school
   3. High school
   4. Vocational training
   5. Bachelor’s
   6. Master’s
   7. PhD

**Below, you will find a number of sentences that describe how you relate to and feel about yourself and others. Read each one and indicate to what extent you feel each sentence is true of you using the scale provided.**

| 1 | 2 | 3 | 4 | 5 |
| --- | --- | --- | --- | --- |
| Not at all |  |  |  | Extremely |
|  |  |  |  |  |

1. I try hard to work on my reputation (in my relationships with others).
2. I do not consider what others say about me.
3. I wish to have a good reputation.
4. I am rarely concerned about my family’s reputation.
5. If my family’s reputation is not good, I feel very bad.
6. I find it difficult if others paint an incorrect image of my family.
7. I care about everyone’s reputation.
8. I am rarely concerned about others’ reputation.
9. I try hard to preserve everyone’s reputation (in my interactions with others).

**Below, you will find a number of sentences that describe how you relate to others. Read each one and indicate to what extent you feel each sentence is true of you using the scale provided.**

| 1 | 2 | 3 | 4 | 5 |
| --- | --- | --- | --- | --- |
| Not at all |  |  |  | Extremely |
|  |  |  |  |  |

1. I will sacrifice my self-interest for the benefit of my group.
2. My relationships with others are more important than my personal accomplishments.
3. I will stay in my group if they need me, even when I am not happy with the group.
4. I stick with my group even through difficulties.
5. I try to abide by customs and conventions at university.
6. I help people I know, even if it is inconvenient.
7. I should be judged on my own merit.
8. I am comfortable being singled out for praise and rewards.

**Post-Test Questionnaire**

**This scale consists of a number of words that describe different feelings and emotions. Read each item and then mark the appropriate answer in the space next to that word. Indicate to what extent you currently feel ______; use the following scale to record your answers.**

| 1 | 2 | 3 | | 4 | 5 | |
| --- | --- | --- | --- | --- | --- | --- |
| Not at all |  |  | |  | Extremely | |
|  |  |  | |  |  | |
| _____ Angry | | | _____ Embarrassed | | |  |
| _____ Happy | | | _____ Confident | | |  |
| _____ Ashamed | | | _____ Mad | | |  |
| _____ Proud | | | _____ Cheerful | | |  |
| _____ Humiliated | | | _____ Respected | | |  |

**Below, you find a number of statements about various actions you may or may not want to take right now based on your experience playing Cyberball. Please indicate using the scale provided how much you want to …**

| 1 | 2 | 3 | 4 | 5 |
| --- | --- | --- | --- | --- |
| Not at all |  |  |  | Extremely |

1. … hurt the other players.
2. … have a chat with the other players.
3. … swear at the other players.
4. … meet the other players.
5. … stay away from the other players.
6. … avoid the other players in real life.

| *For each question, please circle the number to the right that best represents the* ***feelings*** *you experienced* ***DURING THE GAME.*** | *Not at all* |  |  |  | *Extremely* |
| --- | --- | --- | --- | --- | --- |
| I felt liked | 1 | 2 | 3 | 4 | 5 |
| I felt rejected | 1 | 2 | 3 | 4 | 5 |
| I felt humiliated | 1 | 2 | 3 | 4 | 5 |
| I felt ridiculed | 1 | 2 | 3 | 4 | 5 |
